# Electroconductive Photo-Curable PEGDA-Gelatin/PEDOT:PSS Hydrogels for Prospective Cardiac Tissue Engineering Application

**DOI:** 10.3389/fbioe.2022.897575

**Published:** 2022-06-24

**Authors:** Daniele Testore, Alice Zoso, Galder Kortaberria, Marco Sangermano, Valeria Chiono

**Affiliations:** ^1^ Department of Mechanical and Aerospace Engineering, Politecnico di Torino, Turin, Italy; ^2^ Department of Chemical and Environmental Engineering, University of the Basque Country (UPV/EHU), Donostia, Spain; ^3^ Department of Applied Science and Technology, Politecnico di Torino, Turin, Italy

**Keywords:** hydrogels, conductivity, photo-crosslinking, PEGDA, gelatin, PEDOT:PSS, cardiac, tissue engineering

## Abstract

Electroconductive hydrogels (ECHs) have attracted interest for tissue engineering applications due to their ability to promote the regeneration of electroactive tissues. Hence, ECHs with tunable electrical and mechanical properties, bioactivity, biocompatibility and biodegradability are demanded. In this work, ECHs based on photo-crosslinked blends of polyethylene glycol diacrylate (PEGDA) and gelatin with different PEGDA:gelatin ratios (1:1, 1.5:1 and 2:1 wt./wt.), and containing poly (3,4-ethylenedioxythiophene):poly (styrene sulfonate) (PEDOT:PSS) (0.0, 0.1, 0,3 and 0.5% w/v%) were prepared. Main novelty was the use of gelatin as bioactive component and co-initiator in the photo-crosslinking process, leading to its successful incorporation in the hydrogel network. Physical properties could be modulated by the initial PEGDA:gelatin weight ratio. Pristine hydrogels with increasing PEGDA:gelatin ratio showed: (i) an increasing compressive elastic modulus from 5 to 28 kPa; (ii) a decreasing weight loss from 62% to 43% after 2 weeks incubation in phosphate buffered saline at 37°C; (iii) reduced crosslinking time; (iv) higher crosslinking density and (v) lower water absorption. The addition of PEDOT:PSS in the hydrogels reduced photo-crosslinking time (from 60 to 10 s) increasing their surface and bulk electrical properties. Finally, *in vitro* tests with human cardiac fibroblasts showed that hydrogels were cytocompatible and samples with 1.5:1 initial PEGDA:gelatin ratio promoted the highest cell adhesion at 24 h. Results from this work suggested the potential of electroconductive photo-curable PEGDA-gelatin/PEDOT:PSS hydrogels for prospective cardiac tissue engineering applications.

## Graphical Abstract

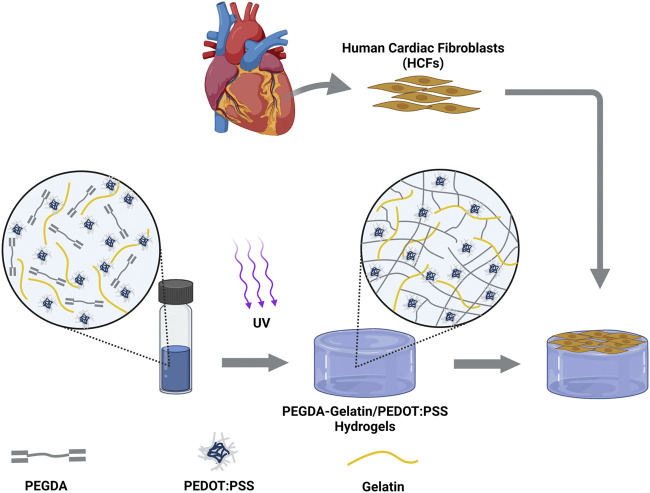

## 1 Introduction

Hydrogels are hydrophilic cross-linked polymeric networks capable of confining and retaining a significant amount of water inside their structure (Hoffman, 2012; Noè et al., 2020). Due to their biomimetic microarchitecture and highly tunable physico-chemical properties they have been increasingly studied in tissue engineering (TE) to mimic soft human tissues, promoting cell attachment, growth and differentiation (Hoffman, 2012; Cavallo et al., 2017; Rogers et al., 2020). Considering that the function of electroactive tissues in the body, such as cardiac, neural and muscle tissue, depends on intra-cellular or extra-cellular electrochemical signaling between cells, TE scaffolds interacting with those tissues should be designed with electroconductive properties (Mehrali et al., 2017; Rogers et al., 2020). However, most hydrogels are typically constituted by non-conductive materials, therefore showing poor electrical properties (Wu et al., 2016).

Electroconductive hydrogels (ECHs) are a class of smart biomaterials that merge the electrical properties of intrinsically conductive components with highly hydrophilic and biocompatible hydrogel networks (Guiseppi-Elie, 2010). ECHs are currently studied for several biomedical applications, including biosensors (Ren et al., 2019; Lee et al., 2020), drug release (Qu et al., 2018; Chen et al., 2021) and tissue engineering (Roshanbinfar et al., 2018; Heo et al., 2019). Several types of conductive dopants have been combined with hydrogels networks to tune their electrical properties while showing biomimetic characteristics respect to the target tissue (Min et al., 2018). Particularly, metal nanoparticles and carbon-based nanomaterials have been widely employed to develop ECHs for tissue engineering application, obtaining highly conductive scaffolds able to improve electrical cell-to-cell communication and therefore to promote the formation of electroactive engineered tissues. However, these conductive materials are typically nonbiodegradable, they could induce oxidative stresses and long-term cytotoxicity thus hindering their clinical application (Allen et al., 2010; Sani et al., 2021).

Conductive polymers (CPs), has been considered as a valid alternative to metals or carbon-based materials in the biomedical field, thanks to their biocompatibility and different possible applications (Balint et al., 2014). Different CPs, such as polypyrrole and polyaniline (Xu et al., 2016) have been used to prepare ECHs for tissue engineering. Poly (3,4-ethylenedioxythiophene):poly (styrene-sulfonate) (PEDOT:PSS), based on a polythiophene derivative, presents high electrical conductivity (3×10^5^–5 × 10^5^ mS/cm), high chemical stability and biocompatibility (Balint et al., 2014; Min et al., 2018). The presence of PSS as a doping agent makes the whole polymeric complex easily dispersible and stable in aqueous solution at physiological conditions (Thaning et al., 2010). Previous short-term *in vivo* studies reported the biocompatibility and biodegradation of polyethylene glycol (PEG)-PEDOT:PSS particles intravenously injected in mice (Cheng et al., 2012). Thanks to its characteristics, PEDOT:PSS has been used for the design of different conductive hydrogels for electroactive tissue engineering. ECHs based on collagen, alginate and PEDOT:PSS were designed to promote the maturation and synchronous beating of neonatal rat cardiomyocytes (Roshanbinfar et al., 2018). In further studies, photo-cured gelatin methacryloyl (GelMA)-PEDOT:PSS hydrogels were initially found to support the viability and spreading of 3D encapsulated myoblasts and subsequently served as a bioink for 3D bioprinting of conductive cell-laden constructs (Spencer et al., 2018; Spencer et al., 2019). Other bioprinted conductive hydrogels for TE applications were developed combining PEDOT:PSS with methylcellulose and kappa-carrageenan (Rastin et al., 2020), or with photocurable polyethylene glycol diacrylate (PEGDA) enabling the differentiation of neural stem cells (Heo et al., 2019). Despite the wide application for tissue engineering, ECHs with tunable electrical and mechanical properties, showing also bioactivity, biocompatibility and biodegradability, are still missing.

Photo-curable hydrogels have gained significant interest in recent years, thanks to their fast gelation kinetics and the precise spatiotemporal control of their properties. In the biomedical field, several photo-crosslinkable hydrogels with tunable mechanical and chemical properties have been reported (Choi et al., 2019). Recently, an innovative photo-curable hybrid natural-synthetic hydrogel for biomedical applications has been proposed by Sangermano and co-workers (Cosola et al., 2019; Zanon et al., 2021). Instead of traditional aliphatic or aromatic amines, unmodified gelatin was used as the co-initiator in a Norrish type II photo-initiating system involving PEGDA as the crosslinker and camphorquinone as the photoinitiator. Thanks to the polymerization mechanism, gelatin segments were chemically crosslinked within PEGDA network, providing arginine-glycine-aspartic acid (RGD) active cell-binding sites. Despite the good biocompatibility results reported by the mentioned works, some concerns about the cytotoxicity of camphorquinone are reported in literature (Hoshikawa et al., 2006). Furthermore, both PEGDA and gelatin are non-conductive materials and therefore unsuitable for regeneration of electroactive tissues, such as cardiac muscle.

The aim of this work was the development of electrically conductive hydrogels with tunable electrical, mechanical and bioactive properties, for tissue engineering application. The photo-curable PEGDA-gelatin photo-initiating system was used with Riboflavin (RF) as a biocompatible type II photoinitiator, replacing camphorquinone. Different PEGDA:gelatin weight ratios were tested to tune mechanical properties of hydrogels. Various concentrations of PEDOT:PSS were finely dispersed within PEGDA-gelatin precursor solutions to impart electrical conductivity to the final system. Photorheological, physico-chemical, mechanical, electrical and *in vitro* degradation properties of hydrogels were evaluated. Finally, as a proof of concept for cardiac tissue engineering application, *in vitro* biocompatibility and adhesion tests with human cardiac fibroblasts (HCF) were performed on hydrogels.

## 2 Materials and Methods

### 2.1 Materials

Poly (ethylene glycol) diacrylate (PEGDA, Mn = 700 g/mol), Gelatin from cold water fish skin, Riboflavin 5′-phosphate sodium salt hydrate (RF) and Phosphate buffered saline (PBS, pH 7.4) were purchased from Sigma-Aldrich (Milano, Italy) and used as received without further purification. Clevios PH 1000, water based emulsion (1.0–1.3 w/v%) of poly (3,4-ethylenedioxythiophene) polystyrene sulfonate (PEDOT:PSS) was purchased from Ossila (Sheffield, United Kingdom). Deionized water (DIH_2_O) was obtained by means of a reverse osmosis purification equipment.

### 2.2 Methods

#### 2.2.1 Precursor Formulations and Hydrogels Preparation

Different amounts of PEGDA and gelatin powder, as described in Table 1, were dissolved in DIH_2_O and gently stirred at room temperature until homogeneous mixtures were obtained. Three different PEGDA:gelatin (w/w) ratios were tested with fixed gelatin concentration at 8% (w/v%). The pH of the commercial PEDOT:PSS aqueous dispersion was neutralized with NaOH 1 M solution, as previously suggested (Roshanbinfar et al., 2018) and the concentration of the neutralized dispersion was systematically verified by drying and weighing. Then, as described elsewhere (Spencer et al., 2018; Rastin et al., 2020), sonication in an ice bath for 30 min was applied to the neutralized PEDOT:PSS dispersion to break large aggregates. PEDOT:PSS was slowly added by small volume increments to the PEGDA and gelatin mixture under strong stirring, to reach the desired concentrations, and the resulting solutions were stirred until they became completely homogenous. RF, previously dissolved in DIH_2_O to obtain a 4.2 mM stock solution, was added to the hydrogel precursor solutions to reach the final compositions. The final concentration of RF in the solution, was fixed at 0.2 mM for all tested formulations. Finally, the precursor formulations, both pristine (without PEDOT:PSS) and doped (with PEDOT:PSS) were sonicated at 25°C for 30 min to further promote homogeneous dispersion of PEDOT:PSS (Spencer et al., 2018).

**TABLE 1:** Compositions of the final solutions exploited for the preparation of the different PEGDA-gelatin/PEDOT:PSS hydrogels, which corresponding codes are reported in the first column.

Code for Crosslinked hydrogels	PEGDA:GELATIN (wt./wt.)	PEDOT:PSS (w/v%)
P1G1	1	0
P1G1P0.1	1	0.1
P1G1P0.3	1	0.3
P1G1P0.5	1	0.5
P1.5G1	1.5	0
P1.5G1P0.1	1.5	0.1
P1.5G1P0.3	1.5	0.3
P2G1	2	0
P2G1P0.1	2	0.1
P2G1P0.3	2	0.3

For 1.5 and 2 PEGDA:gelatin (w/w) ratios, the formulations containing 0.5% w/v of PEDOT:PSS were not prepared due to gelatin precipitation caused by their reduced water content.

Photo-cured PEGDA-gelatin and PEGDA-gelatin/PEDOT:PSS hydrogels were obtained pouring the precursor solutions within homemade silicone molds and exposing them to UV light (Hamamatsu LC8 lamp) for 300 s. The energy dose (70 mW/cm^2^, unless otherwise specified) was periodically checked by means of a EIT POWERPUCK II radiometer (EIT LLC, Leesburg, United States). Unless otherwise stated, hydrogel samples were prepared with rectangular shape (12 mm × 5 mm) and proper thickness (1 mm) for obtaining a complete depth of curing for all the formulations.

#### 2.2.2 Photorheology

Real-time photorheology was performed to investigate photopolymerization kinetics of the UV-cured hydrogels. All rheological experiments were performed on an Anton PAAR Modular Compact Rheometer (Physica MCR 302, Graz, Austria) in parallel-plate mode (25 mm diameter, 0.2 mm of gap) at 37 °C, to avoid any temperature-related physical gelation of gelatin. Preliminary amplitude sweep measurements were carried out at constant shear frequency of 1 Hz to evaluate the linear viscoelastic region of the solutions. Subsequently, photorheological measurements were performed at the same shear frequency and 1% strain amplitude, providing the light with an Hamamatsu LC8 lamp equipped with an optical fiber precisely positioned under the bottom quartz plate. The irradiating light (60 mW/cm^2^ of intensity, periodically checked by radiometer) was turned on after 60 s to allow the stabilization of the system before the onset of the photopolymerization. The evolution of the storage modulus (G′) during the time was recorded to evaluate the polymerization kinetics. Sol-gel phase transition was further evaluated by recording the cross-over points (i.e. time point where G’’/G’ = tan δ = 1).

#### 2.2.3 Fourier Transform Infrared Spectroscopy

The surface chemistry of samples was analyzed by attenuated total reflectance-infrared spectroscopy (ATR-FTIR). Crosslinked samples were dried overnight, then immersed in DIH_2_O at 37 °C for 24 h in order to release the unreacted fraction and finally dried again. Spectra were recorded on a Thermo Scientific Nicolet iS50 FTIR Spectrometer (Milano, Italy) equipped with a diamond crystal ATR accessory. For each sample, ATR spectra were collected in the 4,000–450 cm^−1^ wavenumber range with a resolution of 4 cm^−1^. The analysis was carried out on three different areas for both the top and bottom sample side. Spectra of gelatin and PEGDA were also taken as reference.

#### 2.2.4 Gelatin Release

Release of uncrosslinked gelatin from cured hydrogels was evaluated by a colorimetric test. Briefly, each dried hydrogels sample was weighed and then immersed in 5 ml of DIH_2_O at 37 °C up to 24 h. At predetermined time points (1, 2, 4, 8 and 24 h), the solution was collected for gelatin release evaluation and fresh DIH_2_O was added to the samples. Gelatin concentration was determined by the BCA protein assay (Smith et al., 1985), by a calibration curve obtained from solutions at known gelatin concentrations. The absorbance of each solution at 562 nm was measured by an UV-Vis spectrophotometer (Varioskan™ LUX, Thermo Scientific, United States). The released gelatin fraction was calculated as follows:

$$Gelatin\;Release\;\left(\%\right)=\frac{{\left[Gelatin\right]}_{supernatant}}{{\left[Gelatin\right]}_{total}}*100$$

#### 2.2.5 Water Uptake

The water uptake of hydrogels was evaluated by a gravimetric method. Briefly, after their preparation, hydrogels were completely dried overnight at room temperature and, then, weighed (W_dry_). Completion of the drying process of hydrogels was gravimetrically assessed by zero change in dry weight of samples after overnight, 24 and 48 h storage. Subsequently, dried hydrogels were immersed in 5 ml of PBS solution per hydrogel at 37 °C for 24 h. At each time step (1, 2, 4, 8 and 24 h) samples were collected and weighed again (W_wet_) after carefully removing the excess of water. The percentage water uptake was calculated according to the following equation:

$$Water\;Uptake\;\left(\%\right)=\frac{W_{wet}-W_{dry}}{W_{dry}}*100$$

#### 2.2.6 Scanning Electron Microscopy

The internal microstructure of photo-cured hydrogels was analyzed by scanning electron microscopy (SEM). After crosslinking, the hydrogels were immersed overnight in PBS at 37 °C in order to reach their equilibrium water content. Samples were then washed three times for 5 min in DIH_2_O, frozen at −20 °C and, then, freeze-dried for 48 h (CoolSafe 4-15L freeze-dryer, Labogene, Scandinavia). Freeze-dried samples were rapidly soaked in liquid nitrogen, then fractured and sputter coated with platinum (Q150T Plus turbomolecular pumped coater, Quorum, United Kingdom). SEM images of fracture sections were taken by a Jeol JCM-6000 Plus benchtop SEM (Peabody, United States) operating at 5 kV. Image analysis software (ImageJ, National Institutes of Health, Bethesda, MD, United States) was used to evaluate the dimension of pores in cross-sectional images of samples.

#### 2.2.7 Compression Test

Mechanical characterization of hydrogels was carried out by compression tests using MTS QTestTM/10 Elite controller (MTS Systems Corporation, Edan Prairie, Minnesota, United States) and TestWorks^®^ 4 software (Edan Prairie, Minnesota, United States). Hydrogel samples were prepared in cylindrical molds (11 mm Ø), curing for 300 s at 150 mW/cm^2^ in order to obtain samples with 2.5 mm thickness. The cylinders were compressed at a constant cross-head displacement rate of 0.5 mm/min until 70% strain. The Young’s Modulus (E) of each sample was calculated as the slope of the initial linear portion, from 0% to 10% strain, of the stress-strain curve.

#### 2.2.8 Electrical Measurements

Electrical properties of hydrogels were evaluated by means of sheet resistance and dielectric spectroscopy measurements. The sheet resistance (R_s_) was evaluated using a conventional four-point probe method (Ossila, Sheffield, United Kingdom). Photopolymerized hydrogel surfaces were cleaned from any uncured residual and completely dried overnight at room temperature. Then, the final thickness (t) of dried films was measured with a caliber, before carrying out the measurement. Conductivity (σ) of samples was calculated according to the following equation:

$$\sigma =\;\frac{1}{R_{s}*t}$$

Dielectric spectroscopy measurements were carried out in a Novocontrol Alpha high resolution analyzer (Novocontrol Technologies GmbH & Co. KG, Montabaur, Germany) over a frequency range between 0,1 Hz and 1 MHz at room temperature. The instrument was interfaced to a computer and equipped with a Novocontrol Novocool cryogenic system (Novocontrol Technologies GmbH & Co. KG, Montabaur, Germany) for temperature control. Photopolymerized hydrogels were dried overnight at room temperature to obtain circular sheets. Samples were placed between the gold platted electrodes (10 mm diameter) in a sandwich configuration.

#### 2.2.9 Weight Loss

Weight loss of hydrogels due to their *in vitro* dissolution/degradation was investigated by gravimetric measurement of their dry weight after incubation in PBS for different times. In detail, after cleaning sample surface from any uncrosslinked residue, prepared hydrogels were completely dried and weighed (*W*_i_). Then the samples were incubated in 5 ml of PBS for each sample at 37 °C up to 14 days, refreshing the buffer solution every 3 days. At defined time intervals (1, 3, 7 and 14 days), samples were collected, accurately washed three times in DIH_2_O for 5 min to remove residual PBS salts, dried and weighed again (*W*_f_). The percentage of weight loss was calculated according to the following equation:

$$Weight\;Loss\;\%=\frac{W_{f}-W_{i}}{W_{i}}*100$$

#### 2.2.10 Hydrogel Cytocompatibility

Extracts derived from hydrogels with different compositions were tested for their cell cytocompatibility. *In vitro* cell tests were conducted using human cardiac fibroblasts (HCFs, PromoCell, Germany) seeded in a tissue culture 96-well at a cell density of 10,000 cells/well in complete fibroblast growth medium 3 (FGM-3, PromoCell) and maintained in a humified incubator at 37 °C, 5% CO_2_.

For each sample, 100 μl of hydrogel solution was prepared by dissolving the different components in FGM-3 and polymerized in circular moulds (11 mm Ø) as previously described. Hydrogels were then rinsed with 4 ml of medium for 1 h, weighed and incubated in a 24-well with 1 ml of FGM-3 per 100 mg of hydrogel at 37 °C for 24 h. Extracts were then collected, filtered through a 0.22 μm syringe filter (polyethersulfone membrane, Carlo Erba, Italy) under a sterile hood and added to HCFs cultures.

After 24 h, extracts were removed, and cell viability was assessed by incubation with CellTiter-Blue^®^ Cell Viability Assay (Promega, United States) for 4 h. Finally, fluorescence intensity was measured with a plate reader at ex/em = 530/590 nm. Results were reported as the average fluorescence intensity value normalized to the control (cells cultured in medium without hydrogel extracts).

#### 2.2.11 Live/Dead Imaging

To investigate cell adhesion on hydrogels, direct contact tests with HCFs were performed and results were analysed by Live/Dead assay. Live/Dead assay quickly differentiates live from dead cells by simultaneously staining the culture with two compounds: green-fluorescent Calcein-AM, which detects intracellular esterase activity of living cells, and red-fluorescent ethidium homodimer-1 (EthD-1), which stains dead cell nuclei.

After preparation, hydrogels were sterilized by 10 min incubation with Ethanol 70% v/v, followed by 30 min UV-C irradiation in a sterile hood and a final rinsing with FGM-3 to remove ethanol residues.

Hydrogels were then cultured with HCFs seeded at a cell density of 25,000 cells/hydrogel. After 24 h cells were rinsed with PBS (ThermoFisher, United States) and tested with the Live/Dead assay (ThermoFisher, United States). Briefly, calcein-AM and EthD-1 were diluted in PBS according to manufacturer instructions. The solution was then added to cells and incubated for 30 min at room temperature in the dark. After incubation, Live/Dead solution was discarded and substituted with FGM-3. Samples were visualized using a fluorescence microscope system Nikon Ti2-E (Nikon Instruments, Japan).

#### 2.2.12 Actin/Nuclei Staining

Cells seeded on hydrogels as described in Par 2.2.11, were fixed in paraformaldehyde 4% w/v% in PBS (PFA, Alfa Aesar) for 30 min, after 24 h and 5 days culture time. Fixed cells were permeabilized with Triton X-100 (Sigma-Aldrich) 0.5% v/v% in PBS for 10 min and blocked with bovine serum albumin (BSA, Sigma-Aldrich) 2% w/v% in PBS for 30 min. Cells were then stained with Phalloidin Green 488 (BioLegend) in BSA 2% w/v% and nuclei were counterstained with DAPI (Sigma-Aldrich). Samples were visualized using a fluorescence microscope system Nikon Ti2-E (Nikon Instruments, Japan).

#### 2.2.13 Data Analysis

At least three parallel samples or three different repetitions for each tested formulation were analyzed in each experiment. *In vitro* cell tests were performed using technical and biological triplicates.

Data are reported as mean ± standard deviation (SD). Statistical differences between experimental groups were determined using one-way ANOVA followed by the Tukey’s post hoc test for multiple comparisons. Origin (Pro), Version 2018 (OriginLab Corporation, Northampton, MA, United States) was used for all analyses and plots.

## 3 Results and Discussion

### 3.1 Investigation of Photopolymerization Process

The photoactivated cross-linking process of PEGDA-gelatin hydrogels, with/without PEDOT:PSS, was investigated through photo-rheology. The photo-gelation process was monitored by recording the variations in the storage modulus (G’) as a function of time.

**FIGURE 1:**
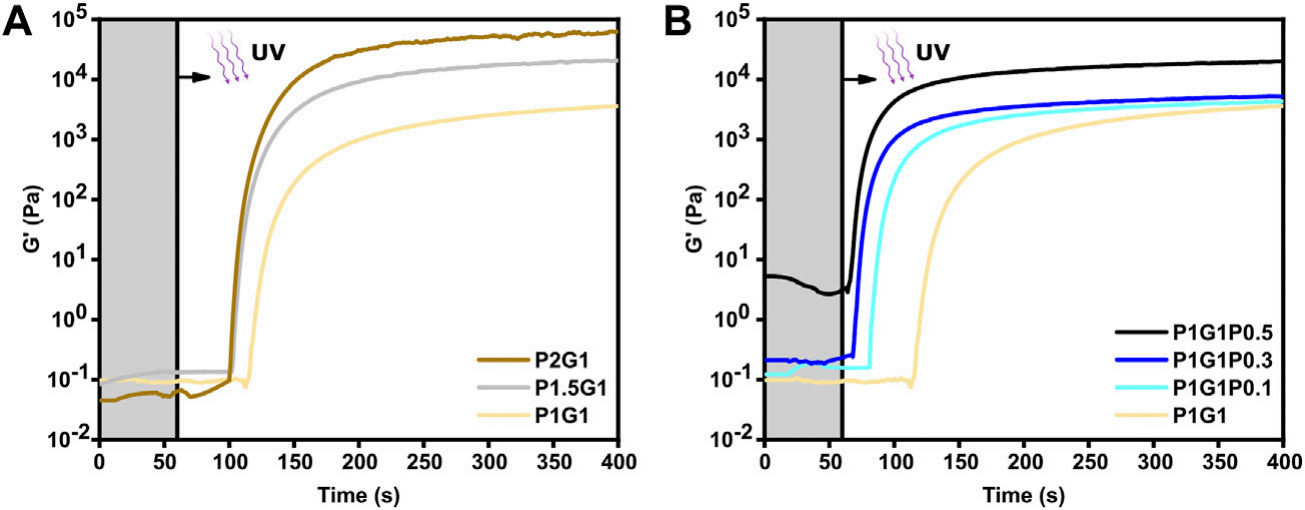
Photopolymerization kinetics of **(A)** pristine hydrogels at different PEGDA:gelatin ratios and **(B)** P1G1 hydrogels with different PEDOT:PSS contents. UV irradiation started after 60 s (grey region).

Initial studies were carried out on the pristine formulations. As shown in Figure 1A, after an initial induction time with unchanged G’ (i.e., the irradiation time required to induce cross-linking), G’ increased as a function of irradiation time, finally reaching a plateau value, suggesting successful photopolymerization for all tested formulations. Therefore, the role of gelatin as a co-initiator in the radical photo-initiating system involving RF as type II photosensitizer was confirmed, in agreement with previous studies (Cosola et al., 2019; Zanon et al., 2021).

As expected, the reactivity of the system as well as its final viscoelastic properties could be finely modulated by varying the ratio between PEGDA and gelatin. Indeed, with increasing PEGDA:gelatin ratio, induction times and cross-over time points decreased. In more detail, induction times of 59, 45 and 43 s and cross-over times of 119, 104 and 102 s were measured for P1G1, P1.5G1 and P2G1 hydrogels, respectively (Supplementary Table S1). While initial slopes of the G′-time curves progressively became steeper, suggesting an increase in the polymerization rates (Figure 1A). Such effects on gelation kinetics were probably due to the progressively higher concentrations of PEGDA in the precursor formulations. Accordingly, higher PEGDA:gelatin ratios resulted in higher final values of G’ (Figure 1A), associated to an increased cross-linking density and decreased overall water content.

Previous reports on photo-crosslinkable hydrogels containing PEDOT:PSS dispersion did not investigate the influence of PEDOT:PSS concentration on the photo-crosslinking kinetics (Spencer et al., 2018; Heo et al., 2019; Spencer et al., 2019; Lin et al., 2021). In this work, photo-rheological tests were exploited to compare photo-crosslinking kinetics of pristine and doped hydrogels. Interestingly, the low PEDOT:PSS concentrations used in this work remarkedly increased photo-crosslinking kinetics. Indeed, average induction times reduced from 59 s for P1G1 samples to only 8 s for P1G1P0.5 samples (Figure 1B). Interestingly, P1G1P0.1 and P1G1P0.3 hydrogels reached similar plateau values of G’ compared to pristine P1G1 hydrogel. The boost effect of PEDOT:PSS on polymerization process could be related to the free-radical formation on PSS chains under UV exposure (Wang et al., 2020). In this case, the presence of PSS probably enhanced the free-radical photogeneration pathway of the RF/gelatin photo-initiating system. Photo-rheological data for pristine and doped P1.5G1 and P2G1 samples evidenced similar trends (Supplementary Figure S1A,B).

The P1G1P0.5 formulation showed a photo-gelation kinetic comparable to that of the other doped formulations, but with a higher starting G’ (Figure 1B). This difference was probably a consequence of the more pronounced molecular interactions between the anionic sulfonic groups of PEDOT:PSS and the cationic amino acids, such as arginine or lysine, present on the gelatin backbone (Spencer et al., 2018; Lee et al., 2020).

### 3.2 Gelatin Cross-Linking Within Hydrogel Network

Previous literature reported that, after taking part in the photo-induced generation of radicals, gelatin remains covalently bonded to the hydrogel network (Cosola et al., 2019; Zanon et al., 2021). In this work, initially the chemical cross-linking of gelatin was qualitatively evaluated by means of infrared spectroscopy. Afterwards, the amount of released gelatin (and, as a difference, the amount gelatin incorporated in the network) was quantified through a colorimetric test. For both analyses, the three different PEGDA-gelatin pristine formulations were compared.

**FIGURE 2:**
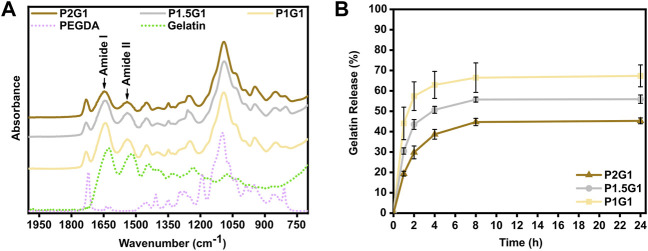
Evaluation of chemical cross-linking of gelatin within hydrogels. **(A)** Attenuated total reflectance-infrared spectroscopy (ATR-FTIR) spectra of pristine hydrogel samples, gelatin powder and non-crosslinked PEGDA in the 2000–700 cm^−1^ wavenumber range. **(B)** Cumulative gelatin release (wt%) from pristine hydrogels at 1, 2, 4, 8 and 24 h in DIH_2_O at 37 °C. Percentages are referred to the total amount of gelatin within the samples.

ATR-FTIR characterization was performed on dried hydrogel previously washed for 24 h in DIH_2_O to release its unreacted soluble fraction. Such washing step was performed at 37 °C to avoid the risk of physical aggregation of gelatin molecules (thermogelation) at lower temperature, ensuring the detection of chemically bond gelatin only. ATR-FTIR spectra of tested samples (P1G1, P1.5G1 and P2G1) showed the presence of the typical absorption bands of gelatin and PEGDA (Figure 2A). Particularly, the two peaks at 1,644 and 1,540 cm^−1^ were attributed to the characteristic amide I (C=O stretching mode) and amide II (N-H bending mode) bands of gelatin, respectively. These results suggested the covalent bonding of gelatin within the hydrogel network as previously reported (Cosola et al., 2019; Zanon et al., 2021). Furthermore, all tested formulations showed the presence of the typical absorption peak of carbonyl groups of PEGDA at 1724 cm^−1^ (C=O stretching mode).

Gelatin release from the pristine PEGDA-gelatin hydrogels incubated in DIH _2_O at 37 °C was then evaluated by BCA assay. For all tested samples, gelatin release started immediately after incubation and increased with time, reaching a plateau at 8–24 h. The total released amount of gelatin decreased with increasing PEGDA:gelatin ratio in the formulations, suggesting that at higher PEGDA amount, more gelatin could remain entrapped in the hydrogel network. After 24 h, P1G1, P1.5G1 and P2G1 released 67 ± 5%, 56 ± 2% and the 45 ± 1% of total gelatin amount, respectively (Supplementary Figure S2B). The statistically significative decreasing amount of released gelatin with increasing PEGDA:gelatin ratio in hydrogels could be due to the increasing reactivity and cross-linking degree of hydrogels, as also suggested by photorheology. Acellular scaffolds for tissue engineering applications are aimed at the initiation of the host tissue regeneration process, exploiting native cell populations *in situ* (Salih, 2009). Therefore, bioactive materials with cell-adhesion active ligands need to be integrated within the scaffolds. PEGDA is biocompatible but biologically inert (Jabbari et al., 2015). On the other hand, arginine-glycine-aspartic acid (RGD) peptide, present along gelatin chains, promotes cell adhesion and spreading via integrin binding (Spencer et al., 2018). After 24 h incubation, PEGDA-gelatin hydrogels retained more than 40% of the initial gelatin content, which may support the process of cell adhesion and proliferation. Importantly, once the cells have adhered, they start to remodel the adhesion substrate, by promoting matrix degradation through the synthesis of degradative enzymes, followed by matrix enrichment/replacement with new extracellular matrix (ECM) molecules.

Furthermore, possible influence of PEDOT:PSS on gelatin incorporation was evaluated. Gelatin release profiles from P1G1P0.5 hydrogels (i.e. the samples with highest PEDOT:PSS content) were analyzed and compared to pristine samples. In doped hydrogels gelatin release kinetics slightly decreased in the first hours (Supplementary Figure S2A), whereas after 24 h (Supplementary Figure S2B) P1G1P0.5 released 61 ± 4% of gelatin with no statistically significant differences respect to P1G1 samples.

### 3.3 Hydrogel Water Uptake and Microstructure

**FIGURE 3:**
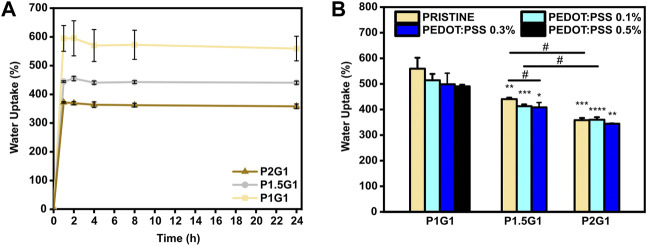
Water uptake of hydrogels: **(A)** Behavior of water uptake percentage vs. time for pristine hydrogels incubated for 1, 2, 4, 8 and 24 h in PBS at 37°C. **(B)** Equilibrium water uptake percentage of hydrogels at 24 h *p < 0.05, **p < 0.01, ***p < 0.001 and ****p < 0.0001 represent significative differences in respect to P1G1 samples. ^#^ *p* < 0.05 represent significative differences for all other comparisons.

Water uptake percentage of PEGDA-gelatin and PEGDA-gelatin/PEDOT:PSS hydrogels was monitored as a function of time by incubating previously dried samples in PBS (pH 7.4, 37 °C). As shown in Figure 3A, pristine hydrogels rapidly reached their maximum water uptake percentage after 2 h incubation, due to their high hydrophilicity. Then, water uptake percentage did not significantly change up to 24 h. Interestingly, hydrogels with progressively higher PEGDA:gelatin ratio showed progressively lower water uptake percentages at 24 h (Figure 3B), with values of 559 ± 43%, 440 ± 6% and 358 ± 8% for P1G1, P1.5G1 and P2G1 samples, respectively. Such trend depends on the higher concentration and crosslinking degree of hydrogels with increasing PEGDA:gelatin ratio, and is in agreement with results from photorheology and gelatin release tests.

The incorporation of increasing PEDOT:PSS amounts in hydrogels the same PEGDA:gelatin ratio did not significantly influence their water uptake (Supplementary Figure S3A,B,C) (Figure 3B). Hence, hydrogels with constant PEDOT:PSS amount continued to display a decreasing trend of the water uptake percentage with increasing PEGDA:gelatin ratio.

Water uptake ability of hydrogels influences cell behavior by affecting different hydrogel properties, such as: (i) hydrogel mesh size, which in turn affects hydrogel permeability (gas and nutrient diffusion) (Lin et al., 2011); (ii) hydrogel stiffness (Bhana et al., 2010), and (iii) electrical properties of conductive hydrogels (Navaei et al., 2019).

In this work, hydrogels showed quick water absorption and, at the same time, slight variations in PEGDA:gelatin ratios significantly changed hydrogels cross-linking degree and, therefore, their water uptake ability. Differently from previous studies which reported an influence of PEDOT:PSS content on hydrogel water uptake ability, water uptake properties of PEGDA-gelatin hydrogels were not affected by the presence of PEDOT:PSS. This result could be the result of a thorough preparation of the hydrogels, by fine dispersion of PEDOT:PSS in the precursor solution, followed by photopolymerization, as described in the Par. 3.1.

**FIGURE 4:**
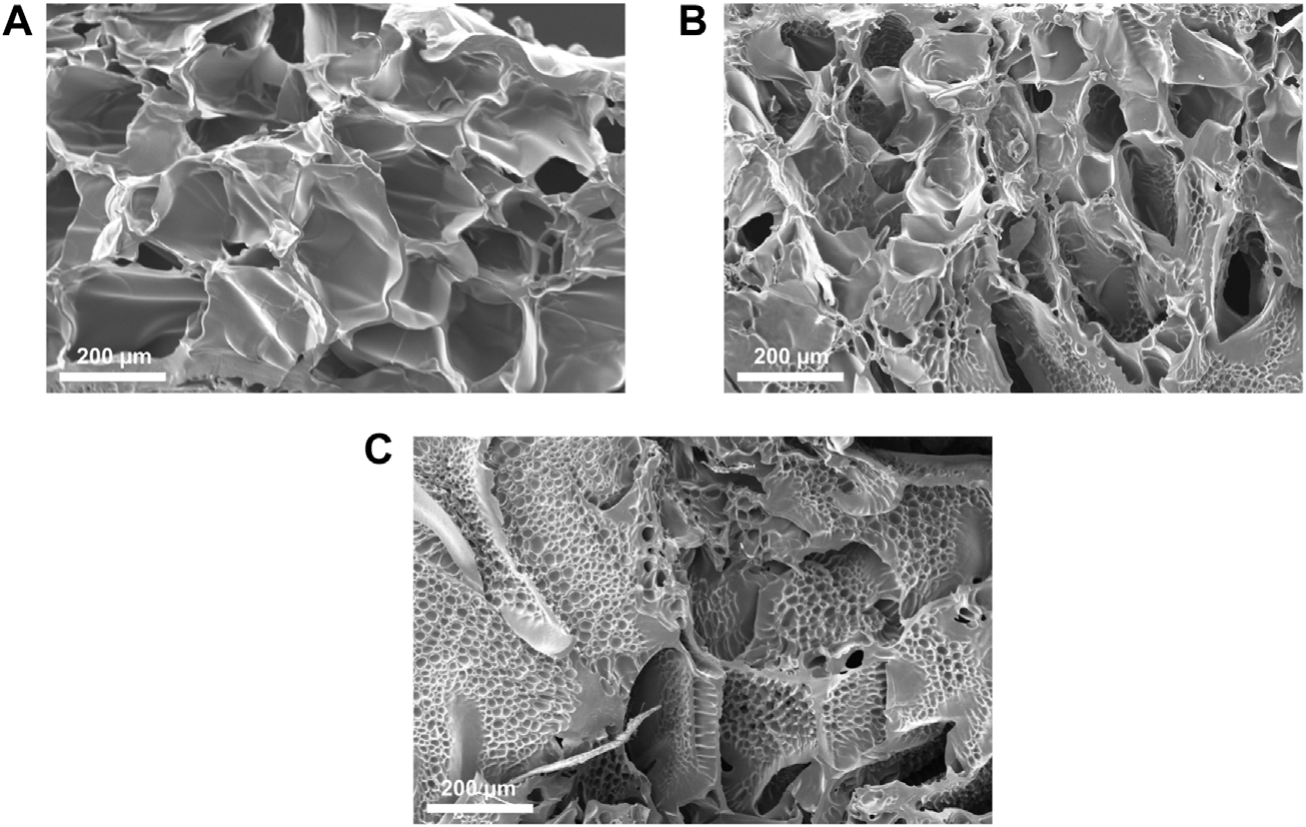
Cross-sectional SEM images of lyophilized **(A)** P1G1, **(B)** P1.5G1 and **(C)** P2G1 pristine hydrogels (scale bars = 200 µm).

Cross-sectional SEM images of freeze-dried PEGDA-gelatin hydrogels (Figure 4) showed that PEGDA:gelatin ratio and hydrogel concentration influenced the microarchitecture of freeze-dried samples. All samples exhibited porous microstructures with interconnected pores and smooth pore walls that would be beneficial for cell migration and proliferation in tissue engineering applications (Parchehbaf-Kashani et al., 2020). P1G1 samples (Figure 4A) showed pores with average size comprised between 30 and 290 μm. P1.5G1 samples (Figure 4B) showed pores in the same size range, together with smaller pores (5–30 µm), which density further increased in P2G1 samples (Figure 4C). Such change in morphology was due to a progressively higher PEGDA content and decreased water content.

Moreover, while other freeze-dried photo-curable hydrogels for tissue engineering applications, based on GelMA or PEGDA have shown unimodal pore distribution (Lin et al., 2011; Spencer et al., 2018), the heterogeneous morphology herein obtained could be beneficial for the stimulation of different cell populations constituting the target tissue (Han et al., 2021). As an example, one previous report suggested that the presence of smaller interconnected pores can favor angiogenesis with negligible fibrosis during cardiac regeneration (Finosh and Jayabalan, 2015).

Additionally, SEM images of pore wall fracture surface of freeze-dried PEGDA-gelatin hydrogels did not show a biphasic morphology, suggesting intermolecular interaction and crosslinking between the two hydrogel components.

As illustrated in Supplementary Figure S4, the addition of PEDOT:PSS did not affect the microstructures of hydrogels as a result of the fine dispersion of the conductive filler in the precursor solution.

### 3.4 Mechanical Characterization

**FIGURE 5:**
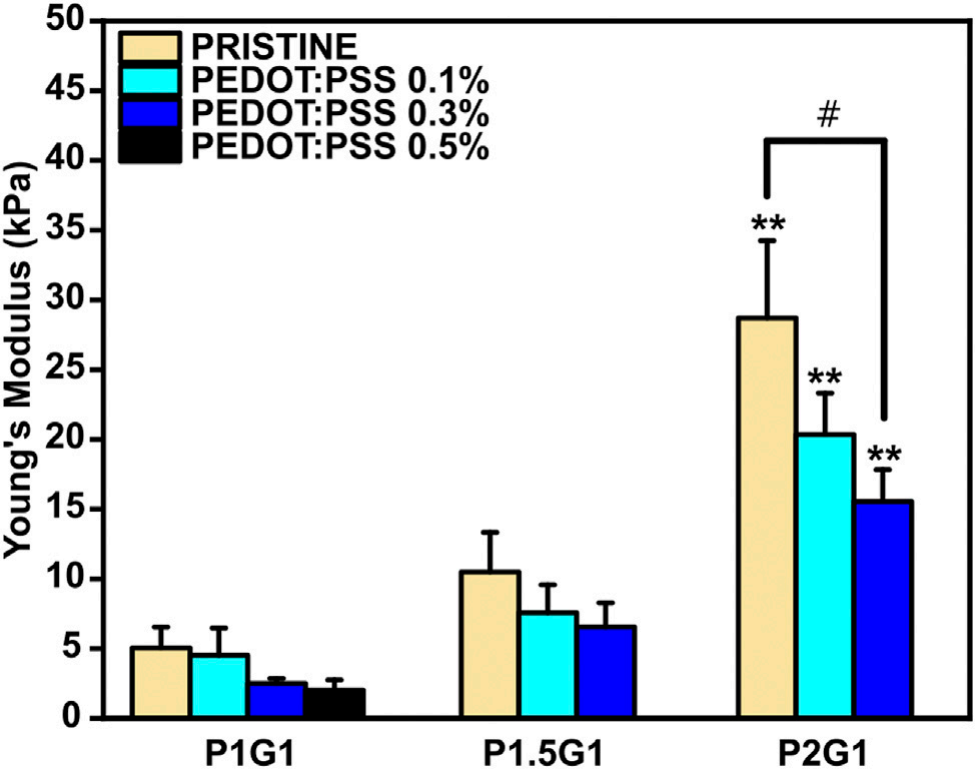
Compressive Young’s Modulus of pristine and doped hydrogels with varying PEGDA:gelatin ratios and PEDOT:PSS contents. **p < 0.01 represent significative differences in respect to P1G1 and P1.5G1 samples. ^#^ *p* < 0.05 represent significative differences for all other comparisons.

It is well established that the three-dimensional hydrophilic microenvironment of hydrogels resembles the natural extracellular matrix (Maharjan et al., 2019). However, hydrogels are also required to display tissue-like mechanical properties for improved integration into the target tissue after implantation (Donnelly et al., 2017; Noshadi et al., 2017). In this work, compressive elastic modulus of PEGDA-gelatin hydrogels was measured from compression stress-strain tests (Supplementary Figure S5). The influence of different PEGDA:gelatin ratios on the final stiffness of photocured hydrogels was studied. As can be observed in Figure 5, elastic modulus of PEGDA-gelatin pristine hydrogels increased from 5.0 ± 1.5 kPa for P1G1, to 10.5 ± 2.8 kPa for P1.5G1 and 28.7 ± 5.5 kPa for P2G1 samples, in agreement with G’ plateau value in photorheological analysis, associated with the different crosslinking degree and water content. According to previous works involving photo-curable PEGDA hydrogels (Mazzoccoli et al., 2009; Noshadi et al., 2017; Wang et al., 2018; Choi et al., 2019), in this work the initial composition of the formulation (i.e., PEGDA:gelatin ratio) allowed a fine control of hydrogel mechanical properties, together with bioactive cues integration, provided by gelatin cross-linking. Notably, the obtained values of stiffness matched the range of natural soft tissues and organs (≈0.1–1,000 kPa), particularly the one of healthy cardiac tissue (≈10–30 kPa) (Bhana et al., 2010; Liu et al., 2015). Recent reports highlighted the role of controlled physical cues in biomaterials-mediated regenerative approaches aimed at post-infarct myocardial regeneration (Paoletti et al., 2018; Paoletti and Chiono, 2021). Hence, the tunable mechanical properties of the herein developed hydrogels make them highly interesting for further investigations in cardiac regenerative approaches.

The presence of PEDOT:PSS caused a slightly reduction in the final Young’s modulus of hydrogels with the same PEGDA:gelatin ratio, with no significant differences except for P2G1 and P2G1P0.3 samples. In agreement with previous reports (Sangermano et al., 2008; Gonzalez et al., 2017; Cortés et al., 2021), the presence of UV light absorbing fillers such as PEDOT:PSS may lead to UV shielding effect reducing photopolymerization kinetics of thick samples at increasing depth from the exposed surface. Therefore, the cross-linking degree of thick cylindrical hydrogels containing PEDOT:PSS was lower on the bottom portion of the samples, affecting the final mechanical properties. Hence, obtaining a complete depth of curing of samples, influence of PEDOT:PSS on final mechanical properties is not expected.

### 3.5 Electrical Characterization

The possibility to tune the electroconductive properties of PEGDA-gelatin/PEDOT:PSS hydrogels by PEDOT:PSS content was thoroughly studied. Indeed, for electroactive tissues regeneration, engineered culture substrates should mediate electrical signaling between cells (Rogers et al., 2020).

The effect of PEDOT:PSS addition on both surface and bulk conductivity of hydrogels was evaluated on dried samples.

**FIGURE 6:**
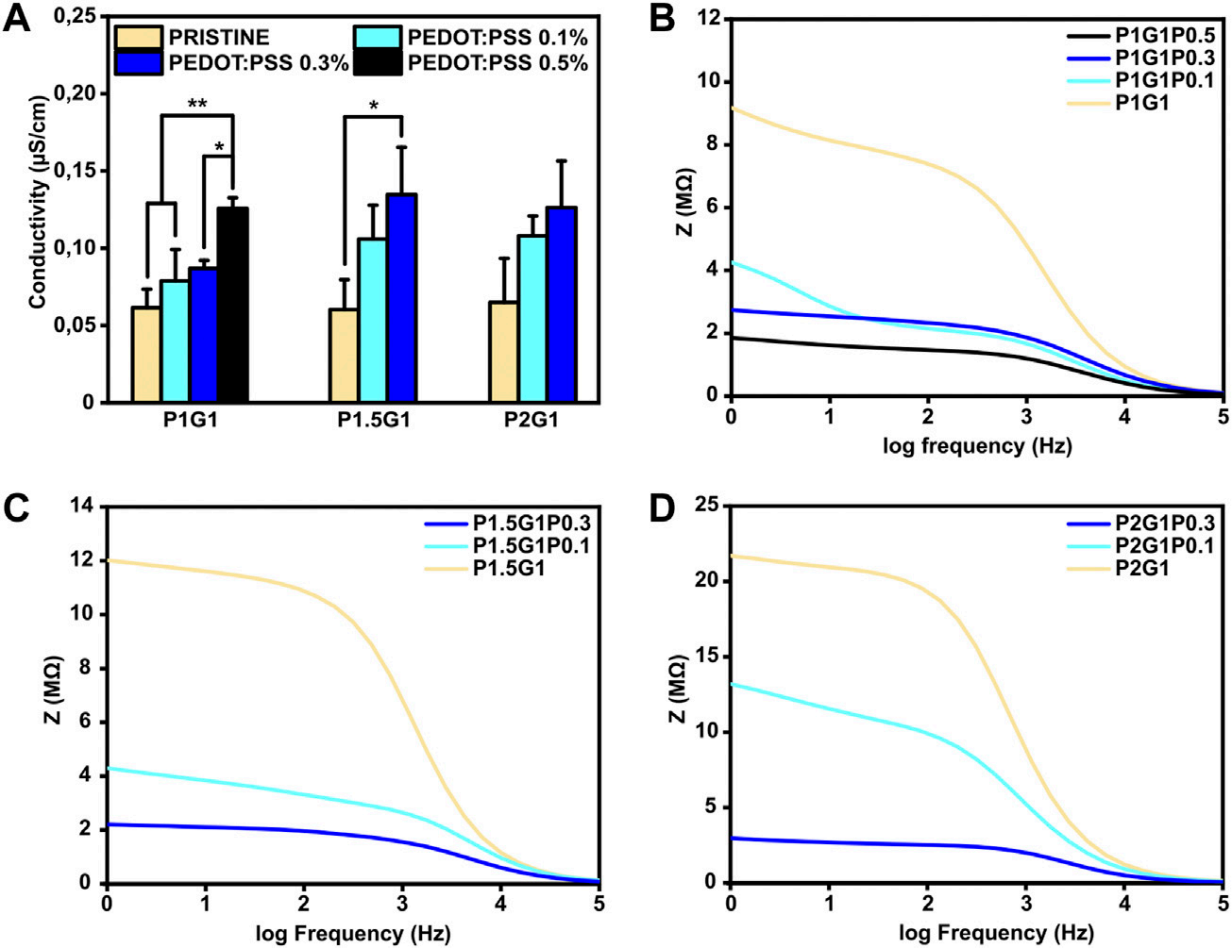
Electrical characterization of PEGDA-gelatin/PEDOT:PSS hydrogels in a dry state. **(A)** Conductivity of all the samples with different PEGDA:gelatin ratio and PEDOT:PSS content measured with a four-point probe. **(B)**, **(C)**, **(D)** Dielectric spectroscopy of P1G1, P1.5G1 and P2G1 samples respectively. *p < 0.05, **p < 0.01.

Firstly, surface conductivities of PEGDA-gelatin/PEDOT:PSS samples were measured by standard four-point probe (Figure 6A). For hydrogels with constant PEGDA:gelatin ratio, conductivity increased as a function of PEDOT:PSS content. Specifically, conductivities raised from 0.06 ± 0.01 μS/cm for the pristine samples to 0.12 ± 0.01 μS/cm for P1G1P0.5 samples and to 0.13 ± 0.03 μS/cm for both P1.5G1P0.3 and P2G1P0.3 samples. Similar range of values were obtained in a previous work reporting the influence of conductive polymer addition on hydrogel conductivities in dry conditions (Guo et al., 2019). Since both gelatin and PEGDA are non-conductive materials, these preliminary results demonstrated a clear contribution of electronic transport introduced by PEDOT:PSS dispersion within the network.

In order to confirm these preliminary results and investigate the bulk electrical properties, dielectric spectroscopy, which applies an alternating current at different frequencies through the entire sample thickness, was performed. Moreover, since physiological currents typically occur in a bidirectional way, this kind of characterization is interesting to test biomaterials for electroactive TE applications (Breukers et al., 2010). As shown in Figures 6B–D, all tested hydrogels exhibited higher impedance at lower frequencies (resistive effect) and lower impedance at higher frequencies (capacitive effect). At 1 Hz, which is the characteristic heartbeat frequency of an adult human in resting conditions, hydrogels containing PEDOT:PSS possessed lower impedance than pristine hydrogels. As an example, for P1G1 samples (Figure 6B), impedance decreased from 9.1 MΩ for pristine formulation to 1.8 MΩ for P1G1P0.5 formulation. At the same time, P1.5G1 and P2G1 samples (Figures 6C,D) showed same trends of impedance variation. All together these results demonstrated the ability of PEDOT:PSS to enhance electroconductive properties of photocurable PEGDA-gelatin hydrogels, suggesting their potential involvement in cardiac tissue engineering.

### 3.6 Hydrogels *in Vitro* Weight Loss

**FIGURE 7:**
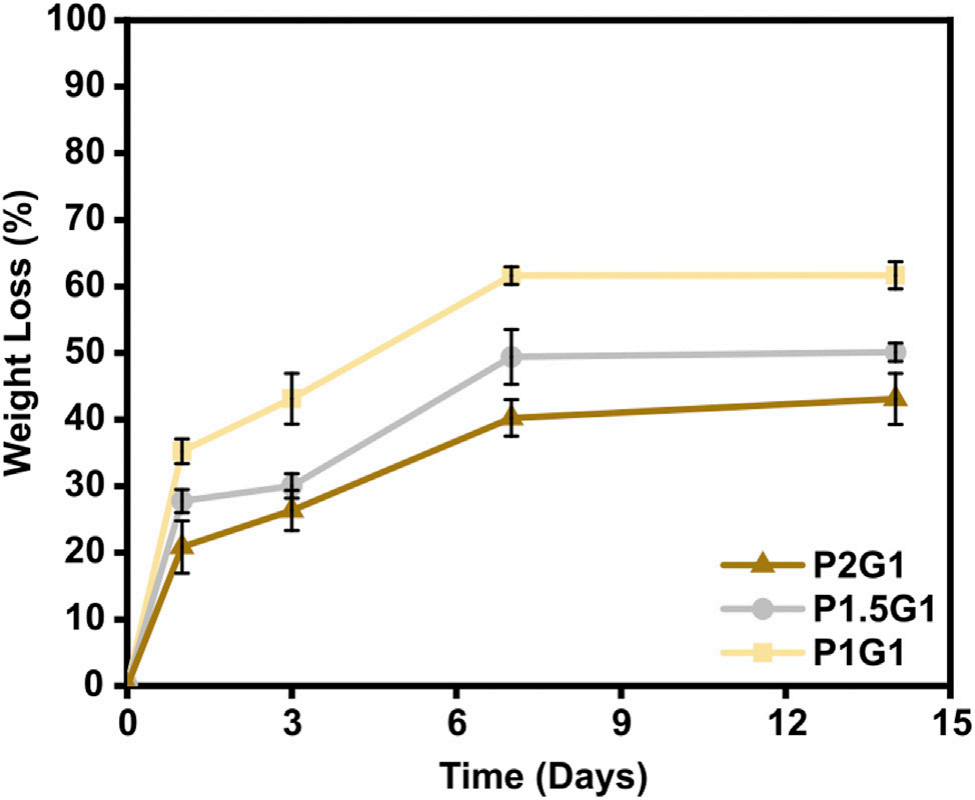
*In vitro* weight loss curves of pristine hydrogels after 1, 3, 7 and 14 days of incubation in PBS at 37°C.

As an important property of hydrogels for TE applications, their *in vitro* weight loss in PBS at 37 °C was evaluated. A controlled rate of weight loss is fundamental to ensure proper integration of the construct with the host tissue before complete degradation (Shie et al., 2020). PEGDA-gelatin hydrogels showed to be stable in PBS for 14 days (Figure 7). After 1 day of incubation, an initial weight loss of 35.2 ± 1.9%, 27.8 ± 1.7% and 20.8 ± 3.9% was measured for P1G1, P1.5G1 and P2G1 hydrogels, respectively, caused by the release of unreacted components. Interestingly, weight loss decreased with increasing PEGDA:gelatin ratio, due to the higher crosslinking degree. At longer times, samples showed a controlled weight loss profile probably due to hydrolytic degradation of gelatin and ester bonds in PEGDA component (Stillman et al., 2020). After 2 weeks, weight loss was 61.7 ± 2.0%, 50.0 ± 1.4% and 43.1 ± 3.8% for P1G1, P1.5G1 and P2G1 hydrogels, respectively.

The influence of PEDOT:PSS on the weight loss profile of hydrogels, as displayed in Supplementary Figure S6A–C, was mostly negligible.

Photo-induced chemical crosslinking process has previously shown to be an effective strategy to improve stability of hydrogels based on natural polymers, which are typically subjected to rapid degradation (Heo et al., 2016). Notably, as a photo-cross-linkable gelatin derivative, GelMA has been widely studied for hydrogel fabrication but its high biodegradation rate has been reported as a drawback (Wang et al., 2018). PEGDA-gelatin hydrogels developed in this work could overcome this limitation thanks to their tunable degradation rate depending on PEGDA:gelation ratio. However, since gelatin is derived from collagen, enzymatic degradation mediated by proteinases could potentially increase biodegradation rate of PEGDA-gelatin hydrogels in the presence of cells.

### 3.7 *In Vitro* Biological Characterization of Hydrogels

In order to evaluate the applicability of developed PEGDA-gelatin/PEDOT:PSS hydrogels as cardiac tissue engineering scaffolds, their *in vitro* cytocompatibility and capability to stimulate cell adhesion was evaluated using HCFs.

**FIGURE 8:**
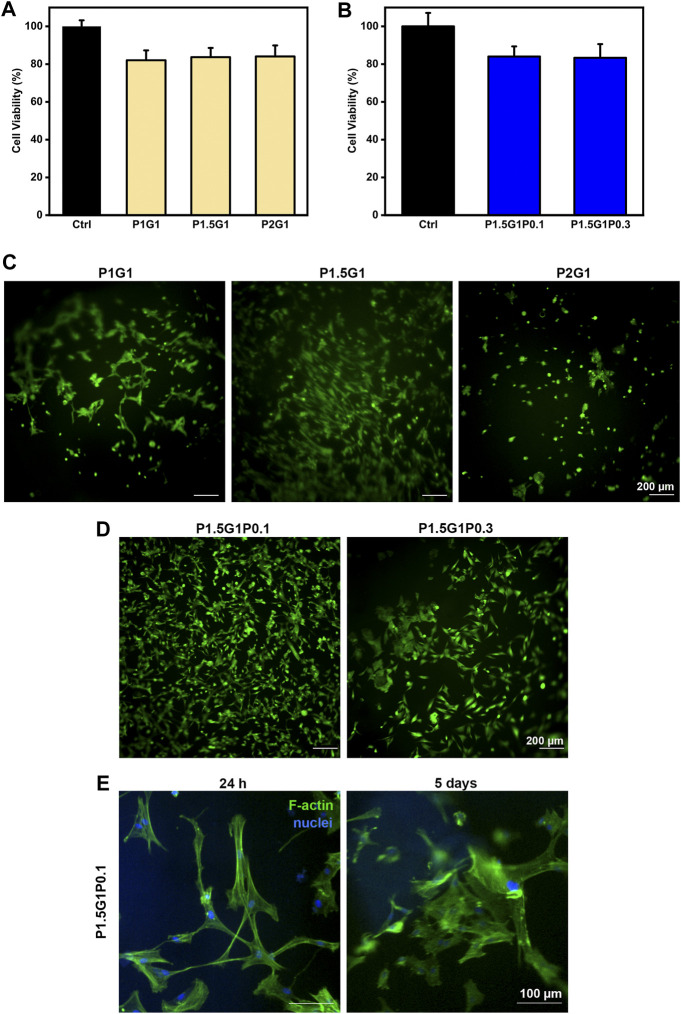
*In vitro* cell characterization of hydrogels. **(A)**, **(B)** indirect cytocompatibility tests of human cardiac fibroblasts, evaluated at 24 h on hydrogel extracts, respectively from **(A)** pristine and **(B)** doped samples. Controls are represented by cells cultured in a medium not containing extracts. **(C)**, **(D)** Viability of HCFs in direct contact with **(C)** pristine and **(D)** doped samples, respectively, after 24 h culture time, evaluated through Live/Dead imaging (scale bars = 200 µm). **(E)** DAPI/F-actin staining of HCFs at 24 h and 5 days for morphological evaluation (scale bars = 100 µm).

The three pristine hydrogel formulations were initially tested. Indirect cytocompatibility tests were conducted following the guidelines of the International Organization for Standardization (ISO 10993–5), incubating cells in medium containing hydrogel extracts (i.e. medium previously in contact with the different PEGDA-gelatin hydrogels). As illustrated in Figure 8A, HCFs cultured with extracts from each pristine formulation showed viability values higher than 80%, compared to the control (i.e. cells cultured in medium not in contact with hydrogels), suggesting non-cytotoxicity according to ISO 10993–5.

Then, HCFs adhesion and viability on pristine hydrogels was demonstrated by Live/Dead staining after 24 h (Figure 8C). Due to its anti-adhesive effect, an increasing content of PEGDA is expected to decrease cell attachment, which on the other hand can be promoted by gelatin (Jabbari et al., 2015). Indeed, cells incubated with P2G1 hydrogels were poorly adherent and showed a round morphology. Interestingly, higher adhesion and spreading of HCFs were observed on P1.5G1 hydrogels than on P1G1. This behavior could be due to the higher gelatin release of P1G1 samples, reducing their bioactivity. Therefore, P1.5G1 hydrogel composition was selected for further characterizations, aimed at the study of conductive PEDOT:PSS influence on HCFs behavior by indirect and direct biocompatibility assays. As shown in Figure 8B, HCFs cultured in the presence of extracts from P1.5G1P0.1 and P1.5G1P0.3 hydrogels showed similar cytocompatibility percentage, respect to HCFs cultured in the presence of extracts from pristine P1.5G1 hydrogel. Live and Dead images of HCFs in direct contact with hydrogels, reported in Figure 8D, showed a similar behavior for P1.5G1P0.1 and P1.5G1 hydrogels, while apparently a reduced amount of viable HCFs was present on P1.5G1P0.3 hydrogels. The presence of a higher PEDOT:PSS amount probably decreased the accessibility of RGD peptide sequences for cell adhesion, slightly reducing the cell-adhesive properties of P1.5G1P0.3 hydrogels. Additionally, P1.5G1P0.1 hydrogels also demonstrated to sustain and maintain cell adhesion and a proper morphology after 24 h post-seeding and up to 5 days of culture, as confirmed by actin/nuclei staining in Figure 8E.

## 4 Conclusion

In this study, PEDOT:PSS was successfully incorporated within photo-curable PEGDA-gelatin networks to obtain electroconductive hydrogels. As important novelty, gelatin was exploited both as bioactive component and co-initiator in the photo-crosslinking process, leading to its successful incorporation in the hydrogel network. For the first time PEGDA-gelatin radical photo-initiating system was optimized for tissue engineering application. By increasing PEGDA:gelatin ratio from 1:1 to 2:1, the photo-crosslinking rate of hydrogels increased and a higher cross-linking density was obtained. Previous studies on ECHs with PEDOT:PSS incorporation did not investigate the influence of the conductive polymer addition on the formation of hydrogel network. In this work, photorheological tests proved that PEDOT:PSS did not hinder the photopolymerization process, but contrarily enhanced the photo-crosslinking kinetics.

The resulting PEGDA-gelatin hydrogels could be finely tuned in their mechanical, water uptake and weight loss properties by simply changing PEGDA:gelatin ratio in the starting formulation. Indeed, by increasing the initial content of PEGDA, stiffer hydrogels with a more dense microarchitecture, a reduced water uptake ability and lower weight loss at physiological conditions were obtained. The elastic compressive modulus of hydrogels was in the range of stiffness values of the native cardiac tissue (≈10–30 kPa). The addition of increasing concentrations of PEDOT:PSS within PEGDA-gelatin samples successfully enhanced their surface and bulk electrical conductivity without remarkedly affecting the physico-chemical properties of hydrogels. Hydrogels were biocompatible and successful incorporation of gelatin within the hydrogels, promoted adhesion of human cardiac fibroblasts. Particularly, P1.5G1 samples proved to have the best balance in terms of gelatin retention, water uptake, weight loss, mechanical and biological properties, promoting HCF adhesion and spreading. PEDOT:PSS containing hydrogels were also biocompatible and, particularly, P1.5G1P0.1 formulation showed superior HCF adhesion ability. In the future, P1.5G1P0.1 hydrogels could be used to promote the maturation and cell-cell interactions of contractile cells (e.g., cardiomyocytes) for cardiac tissue engineering applications.

## Data Availability

The raw data supporting the conclusion of this article will be made available by the authors, without undue reservation.
