# Supplementary material for: Electroconductive Photo-Curable PEGDA-Gelatin/PEDOT:PSS Hydrogels for Prospective Cardiac Tissue Engineering Application
Source: Front Bioeng Biotechnol. 2022 Jun 24;10:897575. doi: 10.3389/fbioe.2022.897575 (PMC9263513; doi:10.3389/fbioe.2022.897575)
Supplement: Supplementary file 1 [file DataSheet1.docx]

Supplementary Material


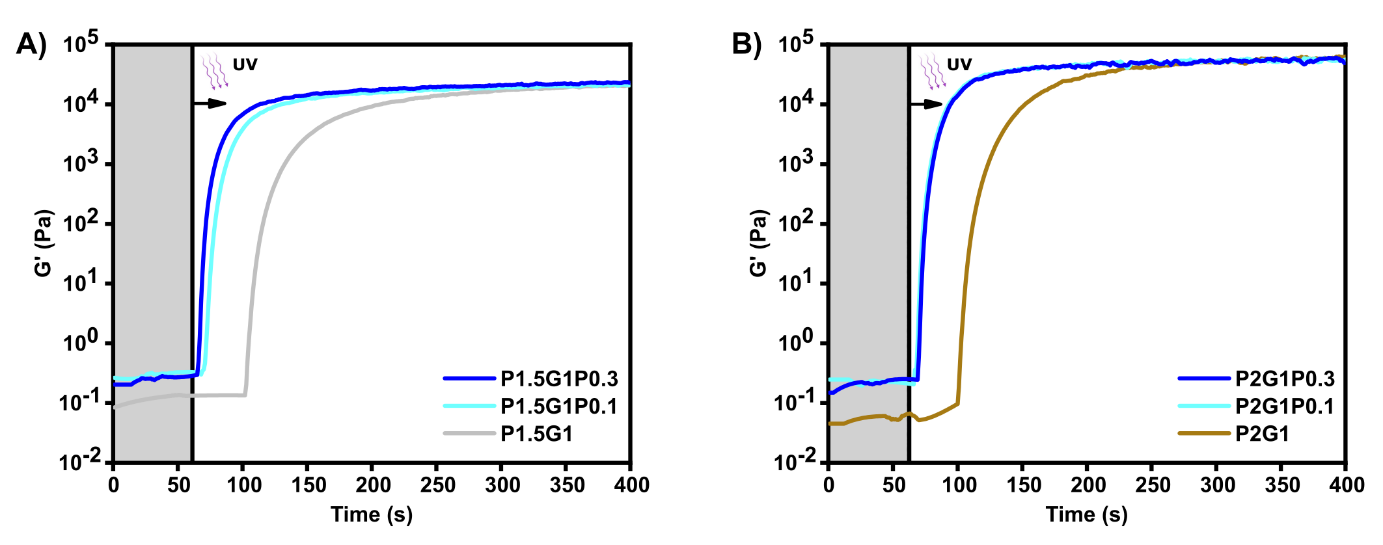


Figure S1 | Photopolymerization kinetics of (A) P1.5G1 hydrogels with different PEDOT:PSS contents, (B) P2G1 hydrogels with different PEDOT:PSS contents. UV irradiation started after 60 s (grey region).

| Code for crosslinked hydrogels | Cross-over point (tan δ = 1) (s) |
| --- | --- |
| P1G1  P1.5G1  P2G1  P1G1P0.1  P1G1P0.3  P1G1P0.5  P1.5G1P0.1  P1.5G1P0.3  P2G1P0.1  P2G1P0.3 | 119 ± 3  104 ± 2  102 ± 1  84 ± 1  73 ± 1  66 ± 1  70 ± 2  69 ± 1  63 ± 1  63 ± 1 |

Table S1 | Cross-over points of the different tested hydrogel formulations. UV irradiation started after 60 s.


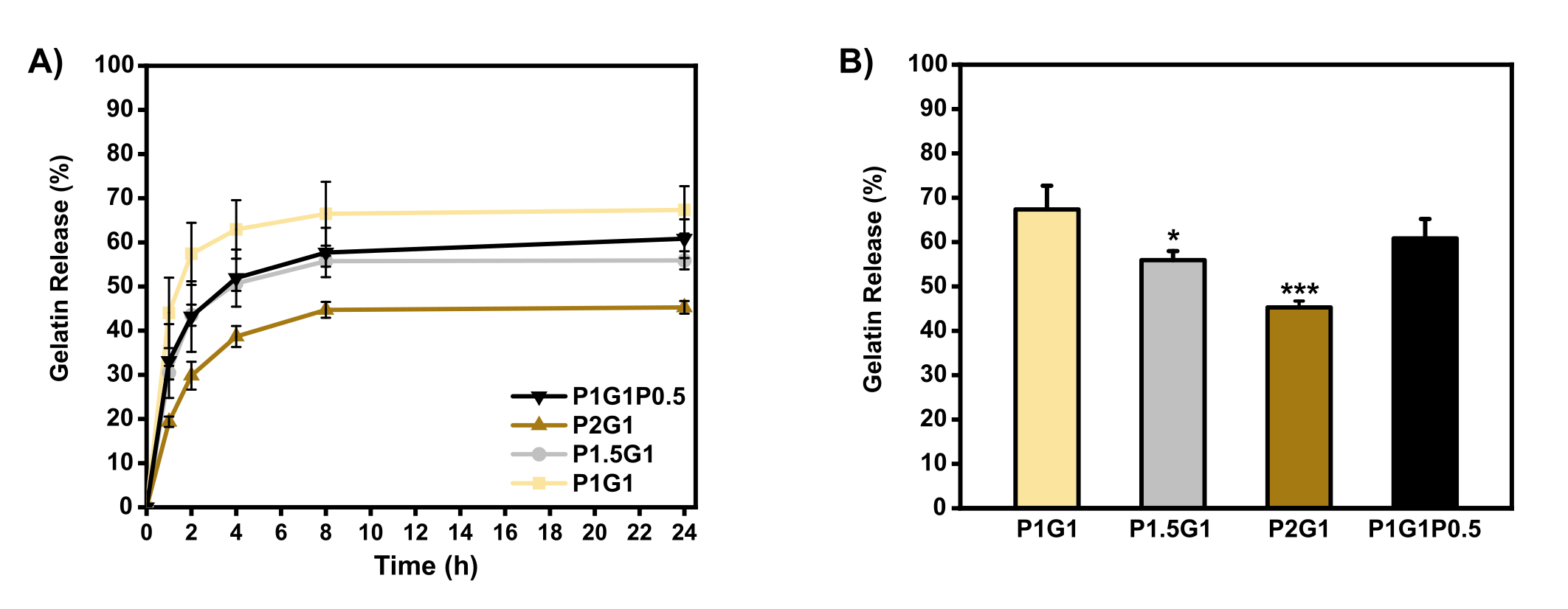


Figure S2 | (A) Cumulative gelatin release (wt. %) from hydrogels at 1, 2, 4, 8 and 24 h in DIH2O at 37 °C. (B) Total amount of gelatin released from hydrogels after 24 h. Percentages are referred to the total amount of gelatin within the samples. * P < 0.05 and *** P < 0.001 represent significative differences in respect to P1G1 samples.


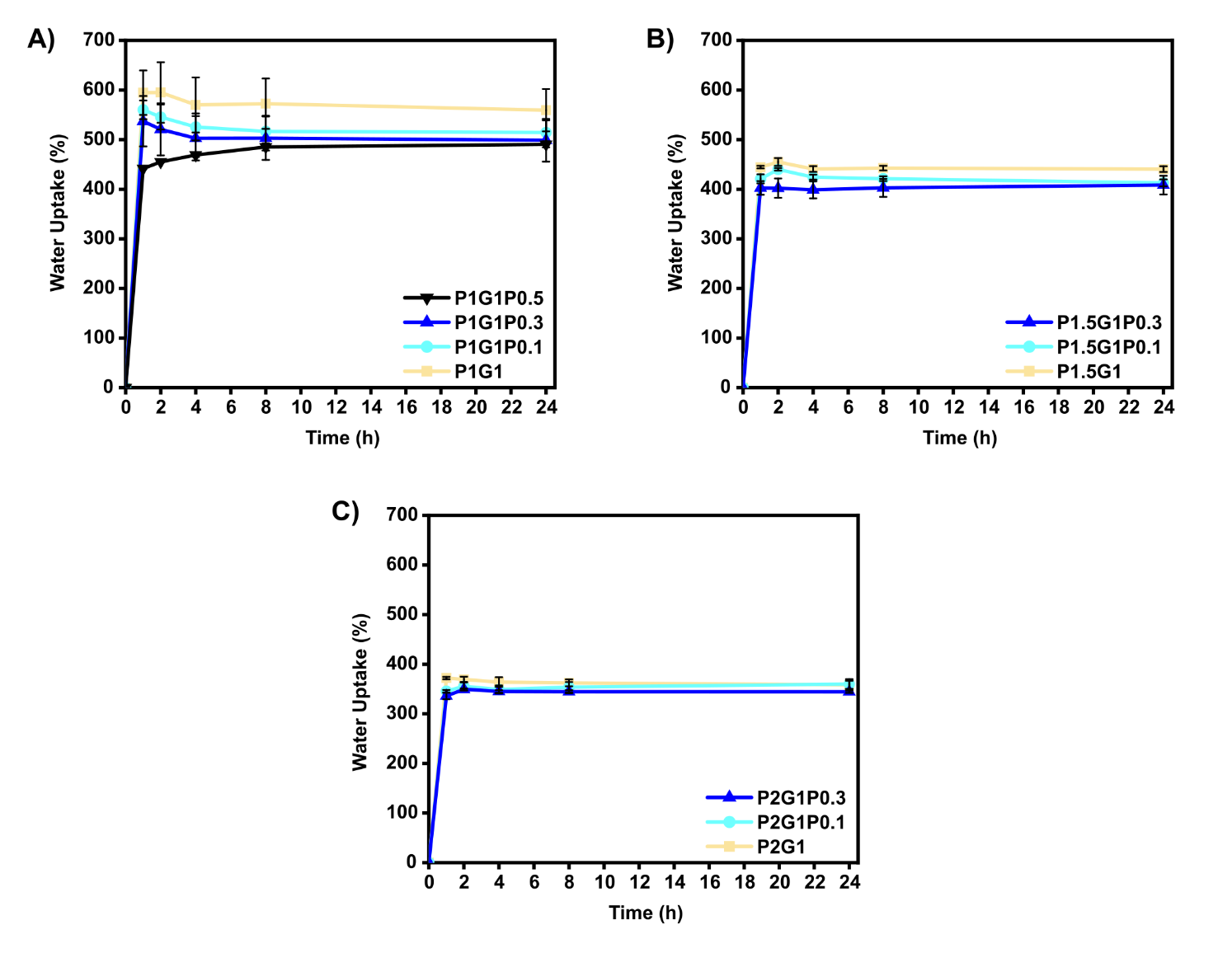


Figure S3 | Behavior of water uptake percentage vs. time for pristine and doped (A) P1G1, (B) P1.5G1, (C) P2G1 hydrogels incubated for 1, 2, 4, 8 and 24 h in PBS at 37°C.


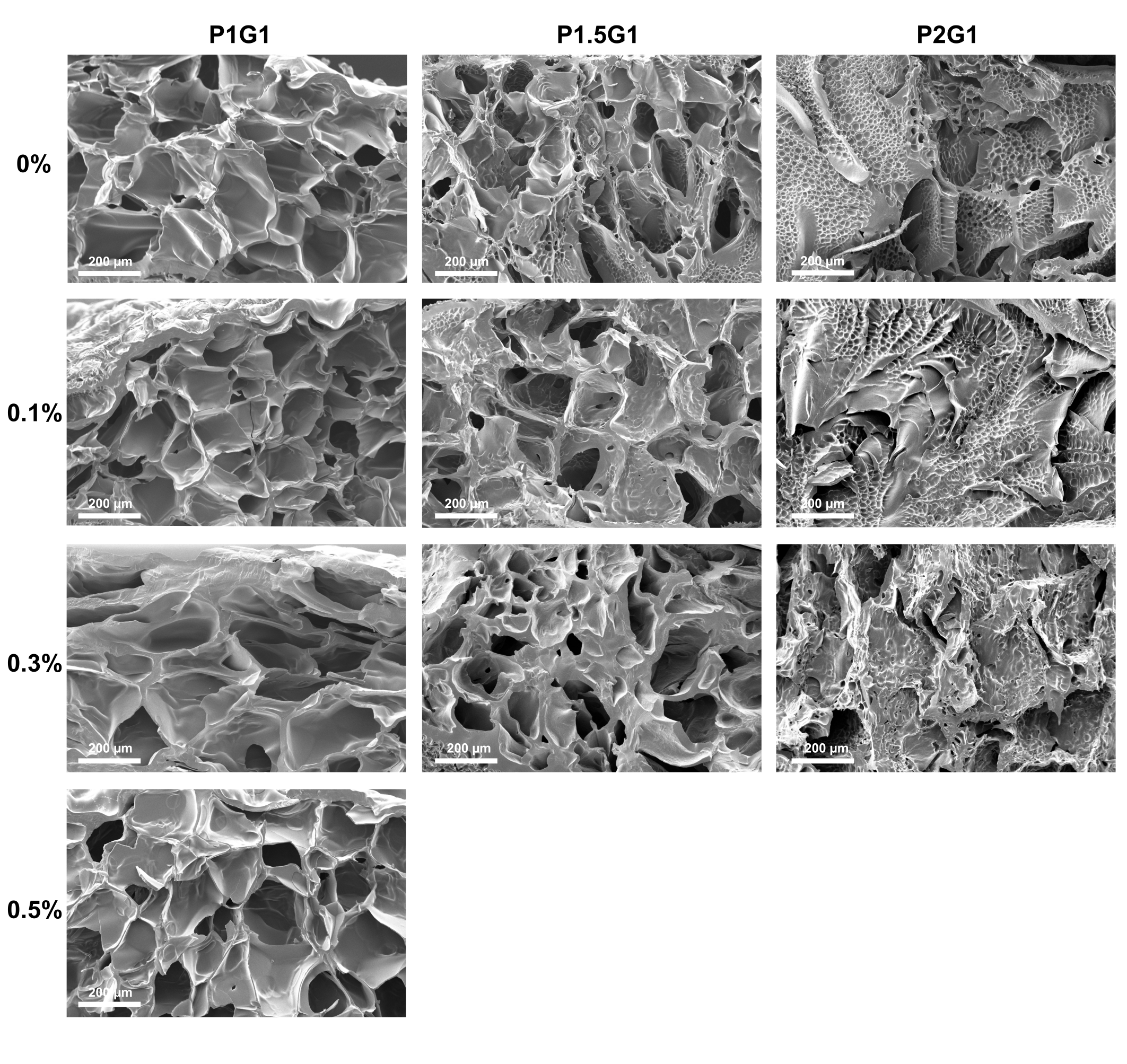


Figure S4 | Cross-sectional SEM images of lyophilized hydrogels. Percentages are referred to PEDOT:PSS content (w/v%) (scale bars = 200 µm ).


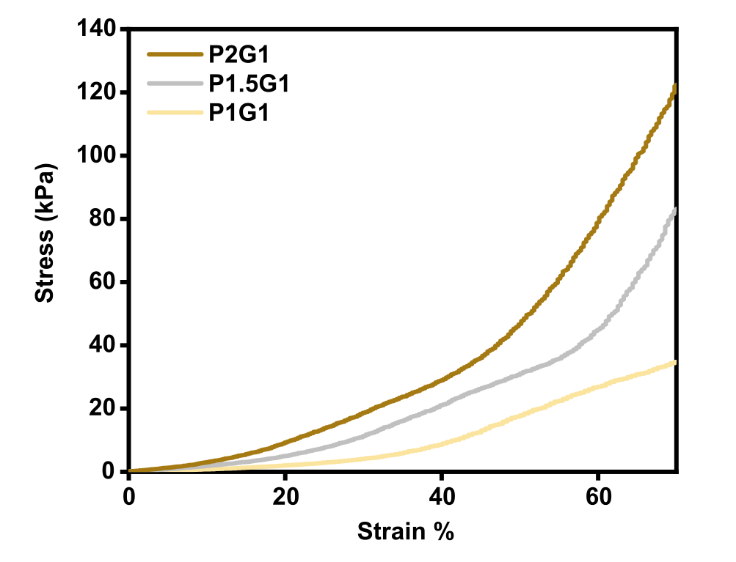


Figure S5 | Compression stress-strain curves of pristine hydrogels with different PEGDA:gelatin weight ratios. Tests were performed in the 0-70% compression strain range.


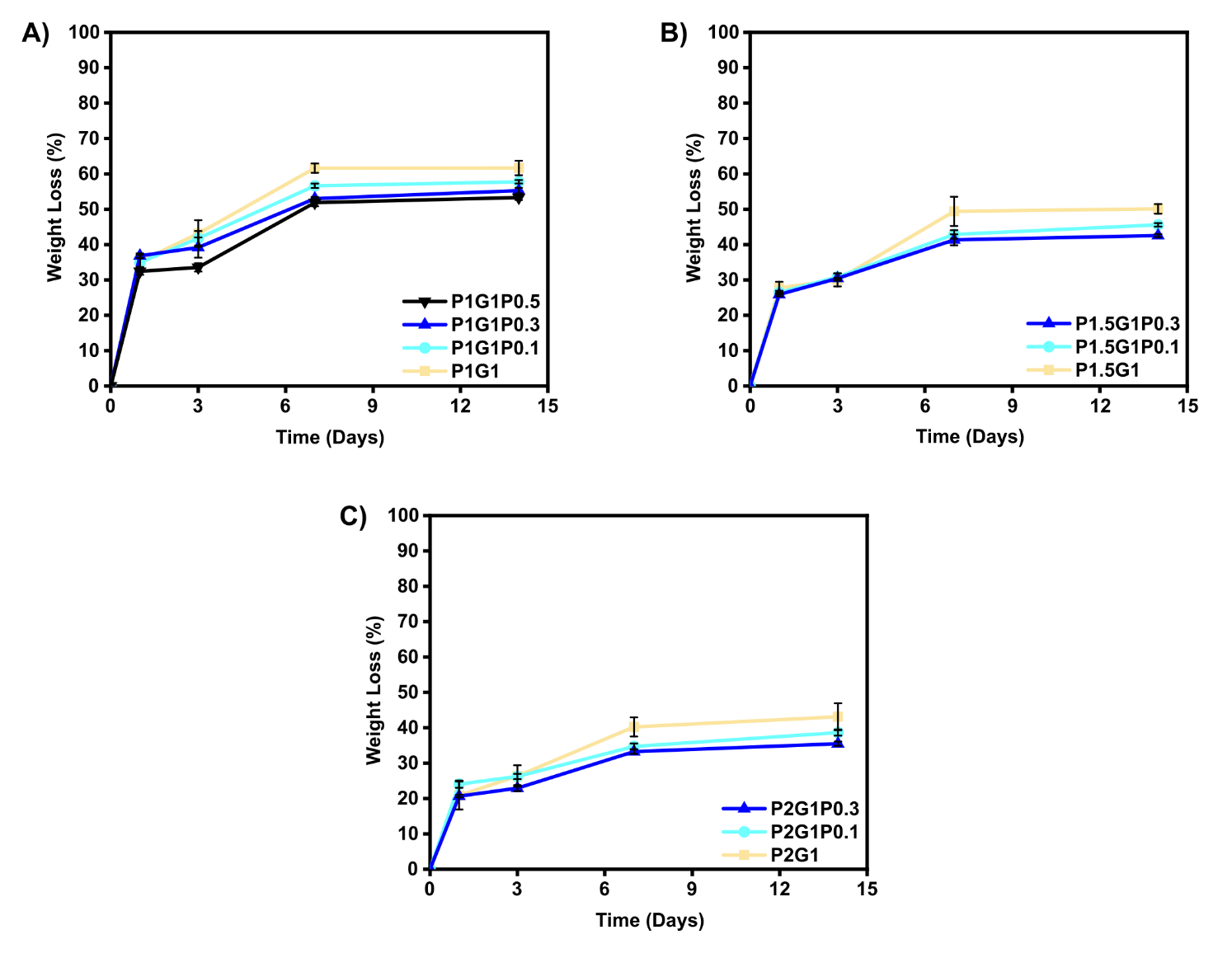


Figure S6 | *In vitro* weight loss curves of (A) P1G1, (B) P1.5G1, (C) P2G1 pristine and doped hydrogels at 1, 3, 7 and 14 days in PBS at 37°C.
